# Association Between Vitamin D and Hyperuricemia Among Adults in the United States

**DOI:** 10.3389/fnut.2020.592777

**Published:** 2020-11-20

**Authors:** Yi-Ying Zhang, Hong-Bin Qiu, Jin-Wei Tian

**Affiliations:** ^1^Department of Cardiology, The Second Affiliated Hospital of Harbin Medical University, Harbin, China; ^2^Key Laboratory of Myocardial Ischemia, Ministry of Education, Harbin Medical University, Harbin, China; ^3^Department of Epidemiology and Biostatistics, School of Public Health, Jiamusi University, Jiamusi, China

**Keywords:** hyperuricemia, vitamin D, cardiovascular disease, NHANES, cross-sectional study

## Abstract

**Background:** Serum uric acid can act as a risk factor for cardiovascular disease (CVD) and as antioxidant defense. Vitamin D deficiency can activate the parathyroid to induce the release of parathyroid hormone, which was thought to increase serum uric acid level, and low vitamin D status may also be associated with risk of CVD. No known studies have explored the association between serum 25(OH) D, vitamin D intake, and HU for the American population.

**Methods:** We extracted 15,723 US adults aged 20–85 years from the National Health and Nutrition Examination Survey (NHANES) in 2007–2014. All dietary intakes were evaluated through 24-h dietary recalls. Multivariable logistic regression analysis was performed to examine the associations after adjustment for confounders.

**Results:** Compared to the lowest quintile (Q1), for males, adjusted odds ratios (ORs) of HU in Q2 to Q4 of serum 25(OH) D levels were 0.78 (95% CI, 0.65–0.93), 0.97 (0.81–1.16), and 0.72 (0.60–0.88); ORs in Q2–Q5 of total vitamin D intake were 0.83 (0.69–0.98), 0.69 (0.58–0.83), 0.66 (0.55–0.79), and 0.59 (0.48–0.71), respectively. In females, OR was 0.80 (0.66–0.97) of serum 25(OH) D for Q3, and ORs in Q5 of total vitamin D intake were 0.80 (0.65–0.98).

**Conclusions:** Our findings indicated that the serum 25(OH) D intakes of dietary vitamin D, supplemental vitamin D, and total vitamin D were inversely associated with HU in males. In females, a lower risk of HU with higher serum 25(OH) D, dietary vitamin D, and total vitamin D intake was found, but with no association between supplemental vitamin D intake and the risk of HU.

## Introduction

Hyperuricemia (HU), which is caused by either overproduction or underexcretion of urate, has been always considered as a precursor of gout due to accumulation of uric acid crystals (1, 2). Several studies have confirmed an association between HU and cardiovascular disease (CVD), serum uric acid can act as a risk factor for CVD and as antioxidant defense (3), and HU has an raised frequency of occurrence among people with high risk of CVD (4). Moreover, recent epidemiologic studies link HU with other diseases such as chronic kidney disease and metabolic syndrome (5, 6). Nowadays, HU is becoming a serious public health problem, many epidemiological studies had shown a growing trend in the prevalence of HU and gout (7, 8).

Vitamin D, the fat-soluble vitamin, is obtained from food, supplements, and sun exposure. The serum concentration of 25-hydroxyvitamin D (25(OH) D) is the best indicator of vitamin D status (9). Several studies have indicated that low vitamin D status may also be associated with risk of CVD (10, 11), chronic kidney diseases (12, 13), and metabolic syndrome (14, 15). Furthermore, vitamin D deficiency can activate the parathyroid to induce the release of parathyroid hormone (16), which was thought to increase serum uric acid level (17–20). As previous clinical research suggested that parathyroid hormone increased the incidence of HU among postmenopausal women (18), parathyroid hormone has significant biologic influence on serum uric acid (17, 20).

No known studies have explored the association between serum 25(OH) D, vitamin D intake, and HU for the American population. Therefore, the purpose of this cross-sectional study is to assess this correlation using a large sample size (15,723 subjects) among both male and female in the United States (US), with a hypothesis that serum 25(OH) D and vitamin D intake is inversely correlated with HU.

## Methods

### Study Populations

Study participants comprised US adults aged 20–85 years who participated in the National Health and Nutrition Examination Survey (NHANES) 2007–2014. NHANES is a continuous survey with data released in two-year cycles of the US civilian, using a stratified, multistage sampling design to attain nationally representative estimates on diet and health indicators. The sample for NHANES is administered by the Centers for Disease Control and Prevention (CDC) (21). NHANES is a publicly available dataset, which resides in the public domain (available on the web at http://www.cdc.gov/nchs/nhanes.htm). Each survey participant completed a household interview and underwent a physical examination at a mobile examination center. Detailed descriptions of NHANES methods are published elsewhere (22, 23). NHANES protocols were approved by the National Center for Health Statistics Research ethics review board, and written informed consent was achieved for all participants (24).

A total of 22,673 adults from 2007 to 2014 aged 20–85 years with uric acid samples constituted the study sample. We excluded pregnant women (*n* = 247); participants taking medications that might affect uric acid metabolism, such as losartan, furosemide, and allopurinol (*n* = 1,404); and those with missing or incomplete essential information on demographic or total nutrient intakes dietary interview (*n* = 5,299). After exclusions, 15,723 adults (7,927 men and 7,796 women) were included in this analysis.

### Study Variables

The major variables included concentrations of uric acid, serum 25(OH) D, and intake of vitamin D. HU was defined as serum uric acid ≥6.0 mg/dL in females and ≥7.0 mg/dL in males (25). Serum uric acid levels were measured using a Beckman UniCel® DxC800 Synchron or a Beckman Synchron LX20 (Beckman Coulter, Inc., Brea, CA, USA) after oxidation of uric acid by uricase to allantoin and hydrogen peroxide. Serum 25(OH)D measurements were performed at the National Center for Environmental Health, CDC, Atlanta, GA, using the DiaSorin radioimmunoassay (RIA) kit (Stillwater MN), and using a standardized liquid chromatography-tandem mass spectrometry (LC-MS/MS) method (26). The intake of total vitamin D, dietary vitamin D, supplemental vitamin D, energy, protein, carbohydrate, magnesium, zinc, fiber, and sugars was obtained from total nutrient intakes provided by the first 24-h dietary recall interviews, which was obtained in-person in the Mobile Examination Center (MEC). Total vitamin D includes both dietary vitamin D and supplemental vitamin D. All patients were interviewed through the first 24-h dietary recall, and a part of patients participated in second dietary surveys by the telephone interviews 3–10 days after the initial recall interview.

Variables that had been demonstrated to be correlated with the vitamin D (serum 25(OH) D and the intake of vitamin D) status as well as HU were included in regression models to control for possible confounding. The covariates including age, race/ethnicity (defined as non-Hispanic white, non-Hispanic black, Mexican American, and others), education (classified into above high school, high school graduation/general educational development (GED), marital status (grouped into married or living with partner, and living alone), weight, height, and blood pressure were conducted following standardized protocol. Body mass index (BMI) was calculated as weight divided by height^2^ (kg/m^2^). Smoking status was grouped as never, current, and former smoker, and participants were divided into never drinkers and current drinkers. Hypertension was identified as systolic blood pressure ≥140 mmHg or diastolic blood pressure ≥90 mmHg (*n* = 3,071), and participants taking antihypertensive medications (*n* = 2,442). Diabetes status was achieved through self-report (*n* = 1,656), and participants taking anti-diabetic medications (*n* = 100). Laboratory analysis covariates included serum creatinine, serum total cholesterol (STC), serum calcium, glucose, serum triglycerides (STG), and high-density lipoprotein cholesterol (HDL-C).

### Statistical Analyses

All statistical analyses were conducted using SAS version 9.4 (SAS Institute Inc., Cary, NC, USA). The continuous variables were presented as median (interquartile range) or mean (standard deviation). Covariates were compared among five groups with differing serum 25(OH) D levels. The categorical variables were characterized by percentage. Differences between continuous variables were assessed by the Wilcoxon rank-sum test and Kruskal–Wallis H test, depending on heteroscedasticity and the skewed distributed data. Differences between categorical variables were evaluated using the chi-square test. Multivariable logistic regression analysis was used to estimate the odds ratio (OR) and 95% confidence interval (CI) of HU, according to the serum 25(OH) D status and vitamin D intake quintile for males and females separately, with the lowest quintile being considered as the references, respectively. Covariates were chosen based on some published studies. Survey weights were not used. Model 1 controlled for age and race/ethnicity. Based on model 1, Model 2 additionally adjusted for drinking status, smoking status, diabetes status, and hypertension status. Based on model 2, Model 3 further controlled for creatinine, total cholesterol, glucose, BMI, HDL-C, triglycerides, serum calcium, magnesium intake, zinc intake, and fiber intake. A sensitivity analysis was undertaken using the second 24-h dietary recall data. *P-*value < 0.05 (two-sided) was considered as statistically significant.

## Results

A total of 15,723 adult subjects were eventually enrolled in this study, which consisted of 7,927 males and 7,796 females. The characteristics of study participants were grouped into five levels, according to the levels of serum 25-hydroxyvitamin D (25(OH) D) quintile, as shown in Table 1. Significant differences were detected across all quintiles of serum 25(OH)D levels for age, race/ethnicity, gender, smoking status, drinking status, education background, marital status, hypertension status, hyperuricemia status, serum uric acid, creatinine, serum total cholesterol (SCT), glucose, body mass index (BMI), high-density lipoprotein cholesterol (HDL-C), serum triglycerides (STG), serum calcium, energy intake, protein intake, carbohydrate intake, magnesium intake, zinc intake, total vitamin D intake, dietary vitamin D intake, supplemental vitamin D intake, fiber intake and sugar intake. Participants with higher serum 25(OH) D levels were more likely to be older, non-Hispanic white, and above high school and have higher serum calcium, creatinine, STC, and intakes of total vitamin D, dietary vitamin D, supplemental vitamin D, dietary fiber intake, dietary magnesium intake, and supplemental intakes of energy, protein, carbohydrate, fiber, magnesium, zinc and sugars and were less likely to be currently smoking and to have hyperuricemia less likely to have lower weight, BMI, and serum uric acid.

**Table 1:** Characteristics of the participants according to the levels of serum 25(OH)D.

**Characteristic**	**Serum 25(OH)D levels (nmol/L)**					***p***
	**<40.8**	**40.8–55.3**	**55.4–67.8**	**67.9–83.8**	**≥83.9**	
	**(*n* = 3,140)**	**(*n* = 3,132)**	**(*n* = 3,160)**	**(*n* = 3,138)**	**(*n* = 3,153)**	
Age (years)	43.00 (30.00, 58.00)	44.00 (31.00, 58.00)	47.00 (34.00, 61.00)	50.00 (36.00, 64.00)	56.00 (40.00, 70.00)	<0.01
Male (*n*,%)	1,499 (47.74)	1,656 (52.87)	1,778 (56.27)	1,684 (53.66)	1,310 (41.55)	<0.01
Race/ethnicity (*n*,%)						<0.01
Non-Hispanic white	569 (18.12)	1,028 (32.82)	1,451 (45.92)	1,876 (59.78)	2,287 (72.53)	
Non-Hispanic black	1,379 (43.92)	672 (21.46)	444 (14.05)	311 (9.91)	275 (8.72)	
Mexican American	565 (17.99)	688 (21.97)	594 (18.80)	384 (12.24)	170 (5.39)	
Others^a^	627 (19.97)	744 (23.75)	671 (21.23)	567 (18.07)	421 (13.35)	
Education background (*n*,%)						<0.01
>High school	1,507 (47.99)	1,568 (50.06)	1,596 (50.51)	1,739 (55.42)	1,881 (59.66)	
High school or GED^b^	762 (24.27)	686 (21.90)	718 (22.72)	710 (22.63)	672 (21.31)	
< High school	871 (27.74)	878 (28.03)	846 (26.77)	689 (21.96)	600 (19.03)	
Marital status (*n*,%)						<0.01
Married or living with partner	1,562 (49.75)	1,837 (58.65)	1,983 (62.75)	2,017 (64.28)	1,952 (61.91)	
Living alone	1,578 (50.25)	1,295 (41.35)	1,177 (37.25)	1,121 (35.72)	1,201 (38.09)	
Drinking status (*n*,%)						<0.01
Never	503 (16.02)	452 (14.43)	401 (12.69)	362 (11.54)	381 (12.08)	
Current	2,637 (83.98)	2,680 (85.57)	2,759 (87.31)	2,776 (88.46)	2,772 (87.92)	
Smoking status (*n*,%)						<0.01
Never	1,771 (56.40)	1,843 (58.84)	1,701 (53.83)	1,663 (53.00)	1,681 (53.31)	
Current	852 (27.13)	678 (21.65)	659 (20.85)	607 (19.34)	571 (18.11)	
Former	517 (16.46)	611 (19.51)	800 (25.32)	868 (27.66)	901 (28.58)	
Weight (kg)	80.80 (68.10, 97.10)	80.70 (68.30, 95.00)	80.30 (68.20, 93.25)	77.80 (66.60, 90.70)	73.60 (63.10, 85.90)	<0.01
Height (cm)	167.10 (159.70, 174.20)	167.25 (160.20, 174.40)	168.35 (161.00, 175.60)	168.30 (161.40, 176.40)	166.50 (159.80, 174.20)	<0.01
BMI (kg/m^2^)	29.05 (24.70, 34.60)	28.90 (25.05, 33.10)	28.12 (24.65, 32.09)	27.20 (24.00, 30.98)	26.20 (23.13, 29.86)	<0.01
Hypertension status (*n*,%)	1,080 (34.39)	991 (31.64)	1,029 (32.56)	1,117 (35.60)	1,296 (41.10)	<0.01
Diabetes status (*n*,%)	371 (11.82)	372 (11.88)	347 (10.98)	328 (10.45)	338 (10.72)	0.26
Hyperuricemia (*n*,%)	798 (25.41)	682 (21.78)	700 (22.15)	637 (20.30)	708 (22.45)	<0.01
Serum uric acid (mg/dL)	5.40 (4.50, 6.50)	5.40 (4.50, 6.40)	5.50 (4.50, 6.40)	5.30 (4.50, 6.30)	5.30 (4.40, 6.20)	<0.01
Serum calcium(mg/dL)	9.40 (9.10, 9.60)	9.40 (9.20, 9.60)	9.40 (9.20, 9.60)	9.40 (9.20, 9.70)	9.50 (9.30, 9.70)	<0.01
Creatinine (mg/dL)	0.82 (0.69, 0.98)	0.82 (0.70, 0.98)	0.85 (0.72, 0.99)	0.87 (0.74, 1.02)	0.88 (0.75, 1.02)	<0.01
Glucose (mg/dL)	93.00 (85.00, 105.00)	93.00 (85.00, 105.00)	93.00 (85.00, 104.00)	93.00 (85.00, 103.00)	92.00 (85.00, 102.00)	0.02
STC (mg/dL)	188.00 (163.00, 218.00)	190.00 (165.00, 218.00)	192.00 (166.00, 219.00)	193.00 (166.00, 221.00)	195.00 (168.00, 221.00)	<0.01
STG (mg/dL)	111.00 (74.00, 174.00)	125.00 (80.00, 197.00)	127.00 (84.00, 198.00)	125.00 (82.00, 192.00)	117.00 (80.00, 178.00)	<0.01
HDL-C (mg/dL)	49.00 (41.00, 60.00)	48.00 (40.00, 58.00)	48.00 (40.00, 59.00)	51.00 (41.00, 62.00)	55.00 (45.00, 67.00)	<0.01
Total vitamin D intake (mcg/day)	2.30 (0.80, 5.20)	3.70 (1.40, 7.90)	5.00 (1.90, 12.30)	7.20 (2.60, 16.70)	13.50 (4.30, 29.20)	<0.01
Dietary vitamin D intake (mcg/day)	2.10 (0.80, 4.50)	3.10 (1.20, 5.90)	3.40 (1.30, 6.50)	3.50 (1.60, 6.60)	3.70 (1.60, 6.70)	<0.01
Supplemental vitamin D intake (mcg/day)	0.81 (4.66)	2.02 (6.21)	4.31 (24.16)	7.60 (26.79)	21.75 (69.42)	<0.01
Energy intake (kcal/day)	1,901.00 (1,402.00, 2,571.50)	1,981.50 (1,455.50, 2,639.50)	1,970.50 (1,452.50, 2,631.00)	1,989.00 (1,498.00, 2,655.00)	1,879.00 (1,416.00, 2,506.00)	<0.01
Supplemental energy intake (kcal/day)	0.91 (7.24)	1.79 (10.27)	2.66 (12.83)	3.86 (16.02)	6.35 (19.18)	<0.01
Protein intake(gm/day)	70.09 (49.53, 97.78)	75.43 (52.75, 103.19)	77.16 (54.69, 104.86)	76.48 (55.24, 103.30)	71.63 (51.66, 98.06)	<0.01
Supplemental protein intake (gm/day)	0.01 (0.14)	0.02 (0.58)	0.06 (1.15)	0.07 (0.97)	0.10 (1.56)	<0.01
Carbohydrate intake (gm/day)	234.21 (165.07, 315.40)	242.42 (176.35, 326.11)	237.65 (173.66, 324.66)	240.78 (176.88, 323.49)	227.44 (166.83, 301.12)	<0.01
Supplemental carbohydrate intake (gm/day)	0.08 (0.93)	0.19 (1.76)	0.28 (2.08)	0.37 (2.19)	0.53 (2.23)	<0.01
Dietary fiber intake (gm/day)	12.50 (8.10, 19.30)	14.70 (9.40, 21.20)	15.10 (9.80, 22.50)	15.40 (10.40, 22.30)	15.40 (10.20, 22.00)	<0.01
Supplemental fiber intake (gm/day)	0.02 (0.39)	0.09 (3.01)	0.08 (0.87)	0.10 (0.86)	0.14 (1.16)	<0.01
Dietary magnesium intake (mg/day)	237.00 (171.00, 330.50)	267.00 (192.00, 361.00)	280.00 (204.00, 377.50)	283.00 (209.00, 381.00)	284.00 (206.00, 381.00)	<0.01
Supplemental magnesium intake (mg/day)	5.69 (41.27)	13.37 (55.71)	19.96 (71.76)	32.75 (89.08)	48.28 (130.34)	<0.01
Dietary zinc intake (mg/day)	8.85 (6.09, 12.95)	9.76 (6.61, 14.06)	10.25 (6.90, 14.53)	10.29 (7.13, 14.87)	9.82 (6.91, 14.32)	<0.01
Supplemental zinc intake (mg/day)	0.81 (4.60)	1.88 (6.22)	3.04 (7.86)	5.11 (10.62)	7.27 (12.51)	<0.01
Dietary sugar intake (gm/day)	97.64 (58.14, 148.04)	101.305 (63.53, 151.91)	98.80 (61.23, 149.52)	101.46 (63.90, 151.54)	95.16 (60.89, 141.94)	0.001
Supplemental sugar intake (mg/day)	0.05 (0.50)	0.13 (0.94)	0.17 (1.29)	0.20 (1.27)	0.24 (1.13)	<0.01

a Other Hispanics and other races including multi-racial participants.

b *General Educational Development*.

The results comparing the vitamin D status and other indicators between HU and non-HU for both sexes are shown in Table 2. For male, serum 25(OH) D levels and intakes of total vitamin D, dietary vitamin D, and supplemental vitamin D indicators were significantly different between HU and non-HU. Compared to the participants without HU, participants with HU had lower serum 25(OH) D levels and lower intakes of total vitamin D and dietary vitamin D. For female, serum 25(OH) D levels and intakes of dietary vitamin D and supplemental vitamin D indicators were significantly different between HU and non-HU. Participants with HU had lower serum 25(OH) D levels and intakes of dietary vitamin D. Patients with HU had higher levels of BMI, serum calcium, creatinine, STC, STG, and glucose and lower level of HDL cholesterol than those participants without HU for both sexes.

**Table 2:** Characteristics of participants with or without hyperuricemia.

**Characteristic**	**Male**		***p***	**Female**		***p***
	**Non-hyperuricemia**	**Hyperuricemia**		**Non-hyperuricemia**	**Hyperuricemia**	
	**(*n* = 6,175)**	**(*n* = 1,752)**		**(*n* =6,023)**	**(*n* = 1,773)**	
Serum 25(OH)D levels (nmol/L)	61.30 (46.30, 76.90)	58.95 (43.60, 75.40)	0.00	62.60 (44.50, 82.40)	61.60 (41.90, 83.20)	0.08
<30	441 (73.87)	156 (26.13)	0.02	518 (71.84)	203 (28.16)	0.00
30–49	1,482 (77.47)	431 (22.53)		1,405 (76.78)	425 (23.22)	
50–125	4,179 (78.38)	1,153 (21.62)		3,861 (77.98)	1,090 (22.02)	
≥126	73 (85.88)	12 (14.12)		239 (81.29)	55 (18.71)	
Total vitamin D intake (mcg/day)	5.20 (2.00, 12.80)	4.20 (1.40, 11.00)	<0.01	4.90 (1.70, 14.30)	4.70 (1.60, 15.80)	0.59
<15	4,913 (77.14)	1,456 (22.86)	0.00	4,576 (77.78)	1,307 (22.22)	0.05
≥15	1,262 (81.00)	296 (19.00)		1,447 (75.64)	466 (24.36)	
Supplemental vitamin D intake (mcg/day)	5.54 (32.66)	4.18 (12.54)	0.03	9.52 (45.39)	9.0323 (24.18)	0.03
Non-user	4,723 (77.31)	1,386 (22.69)	0.06	4,164 (77.96)	1,177 (22.04)	0.12
<15	845 (80.78)	201 (19.22)		769 (76.59)	235 (23.41)	
15–100	566 (78.28)	157 (21.72)		1,012 (74.96)	338 (25.04)	
≥100	41 (83.67)	8 (16.33)		78 (77.23)	23 (22.77)	
Dietary vitamin D intake (mcg/day)	3.70 (1.50, 6.90)	2.90 (1.10, 6.05)	<0.01	3.00 (1.10, 5.60)	2.60 (1.00, 4.90)	0.00
<15	5,825 (77.58)	1,683 (22.42)	0.00	5,860 (77.16)	1,735 (22.84)	0.19
≥15	350 (83.53)	69 (16.47)		163(81.09)	38 (18.91)	
BMI (kg/m^2^)	27.10 (24.00, 30.52)	29.87 (26.60, 34.12)	<0.01	26.92 (23.28, 31.60)	31.90 (27.33, 37.20)	<0.01
Serum calcium (mg/dL)	9.40 (9.20, 9.70)	9.50 (9.20, 9.70)	0.00	9.40 (9.20, 9.60)	9.50 (9.20, 9.70)	<0.01
Creatinine (mg/dL)	0.94 (0.84, 1.06)	1.02 (0.91, 1.17)	<0.01	0.72 (0.63, 0.82)	0.82 (0.72, 0.97)	<0.01
Glucose (mg/dL)	93.00 (86.00, 104.00)	96.00 (88.00, 108.00)	<0.01	90.00 (83.00, 100.00)	96.00 (88.00, 110.00)	<0.01
STC (mg/dL)	187.00 (161.00, 215.00)	194.00 (167.00, 223.00)	<0.01	193.00 (167.00, 220.00)	201.00 (174.00, 230.00)	<0.01
STG (mg/dL)	124.00 (81.00, 196.00)	153.00 (102.00, 240.00)	<0.01	105.00 (71.00, 158.00)	141.00 (95.00, 20,800)	<0.01
HDL-C (mg/dL)	46.00 (39.00, 56.00)	43.00 (36.00, 52.00)	<0.01	56.00 (47.00, 67.00)	51.00 (43.00, 62.00)	<0.01

The association between serum 25(OH) D levels and HU was observed in the multivariable model, as is shown in Table 3. There was an inverse trend between higher serum 25(OH) D levels and risk of HU, after controlling for age, race/ethnicity, drinking status, smoking status, diabetes status, hypertension status, creatinine, SCT, glucose, BMI, HDL-C, STG, serum calcium, magnesium intake, zinc intake, and fiber intake. In males, compared with the lowest quintile (Q1; serum 25(OH) D levels <41.8 nmol/L), the adjusted odds ratios (ORs) of HU in Q2–Q4 were 0.78 (95% CI, 0.65–0.93), 0.97 (95% CI, 0.81–1.16), and 0.72 (95% CI, 0.60–0.88), respectively, and that of Q5 was 0.82 (95% CI, 0.68–1.00); *p* for the trend was 0.0479. In females, compared with Q1 (respondents with serum 25(OH) D levels <39.8 nmol/L), OR was 0.80 (95% CI, 0.66–0.97) for Q3 (55.4–69.3 nmol/L), and *p* for the trend was 0.0977.

**Table 3:** Adjusted odds ratios of hyperuricemia among participants associated with serum 25(OH)D.

		**Serum 25(OH)D levels (nmol/L)**					***p* for trend**
		Q1 (<41.8)	Q2 (41.8–55.4)	Q3 (55.5–66.4)	Q4 (66.5–80.4)	Q5 (≥80.5)	
		(*n* = 1,583)	(*n* = 1,582)	(*n* = 1,579)	(*n* = 1,593)	(*n* = 1,590)	
Male (*n* = 7,927)	Model 1^a^	Reference	0.80 (0.68, 0.95)	0.94 (0.79, 1.11)	0.68 (0.57, 0.82)	0.72 (0.60, 0.86)	<0.0001
	Model 2^b^	Reference	0.79 (0.67, 0.94)	0.93 (0.78, 1.10)	0.67 (0.56, 0.80)	0.71 (0.59, 0.86)	0.0001
	Model 3^c^	Reference	0.78 (0.65, 0.93)	0.97 (0.81, 1.16)	0.72 (0.60, 0.88)	0.82 (0.68, 1.00)	0.0479
		Q1 (<39.8)	Q2 (39.8–55.3)	Q3 (55.4–69.3)	Q4 (69.4–87.2)	Q5 (≥87.3)	
		(*n* = 1,556)	(*n* = 1,561)	(*n* =1,549)	(*n* = 1,562)	(*n* = 1,568)	
Female (*n* = 7,796)	Model 1^a^	Reference	0.85 (0.71, 1.01)	0.72 (0.60, 0.86)	0.71 (0.59, 0.85)	0.67 (0.56, 0.80)	<0.0001
	Model 2^b^	Reference	0.84 (0.71, 1.00)	0.72 (0.60, 0.86)	0.72 (0.60, 0.86)	0.69 (0.57, 0.83)	<0.0001
	Model 3^c^	Reference	0.90 (0.75, 1.09)	0.80 (0.66, 0.97)	0.83 (0.68, 1.01)	0.85 (0.69, 1.05)	0.0977

a Adjusted for age, race/ethnicity;

b adjusted for age, race/ethnicity, smoking status, drinking status, hypertension status and diabetes status;

c *adjusted for age, race/ethnicity, smoking status, drinking status, hypertension status, diabetes status, creatinine, STC, glucose, BMI, HDL-C, STG, serum calcium, magnesium intake, zinc intake, fiber intake*.

There was an inverse trend between higher dietary vitamin D intakes and risk of HU, as is shown in Table 4. In males, compared with Q1 (respondents consuming <1.1 mcg dietary vitamin D daily), the adjusted ORs of HU in Q3 (consuming 2.6–4.5 mcg dietary vitamin D daily), Q4 (consuming 4.6–7.6 mcg daily), and Q5 (consuming 7.7 mcg or greater) were 0.69 (95% CI, 0.58–0.83), 0.68 (95% CI, 0.57–0.82), and 0.57 (95% CI, 0.47–0.69), respectively, *p* for trend < 0.0001. In females, compared with Q1 (respondents consuming <0.9 mcg daily), the OR of HU was 0.81 (95% CI, 0.67–0.98) for Q4 (consuming 3.7–6.2 mcg dietary vitamin D daily), and *p* for trend was 0.0024.

**Table 4:** Adjusted odds ratios of hyperuricemia among participants associated with dietary vitamin D intake.

		**Dietary vitamin D intake (mcg/day)**					***p* for trend**
		Q1 (<1.1)	Q2 (1.1–2.5)	Q3 (2.6–4.5)	Q4 (4.6–7.6)	Q5 (≥7.7)	
		(*n* = 1,619)	(*n* = 1,525)	(*n* = 1,603)	(*n* = 1,578)	(*n* = 1,602)	
Male (*n* = 7,927)	Model 1^a^	Reference	0.92 (0.78, 1.08)	0.72 (0.61, 0.85)	0.73 (0.62, 0.86)	0.62 (0.52, 0.74)	<0.0001
	Model 2^b^	Reference	0.91 (0.77, 1.07)	0.71 (0.60, 0.84)	0.71 (0.60, 0.84)	0.61 (0.52, 0.73)	<0.0001
	Model 3^c^	Reference	0.88 (0.74, 1.05)	0.69 (0.58, 0.83)	0.68 (0.57, 0.82)	0.57 (0.47, 0.69)	<0.0001
		Q1 (<0.9)	Q2 (0.9–2.0)	Q3 (2.1–3.6)	Q4 (3.7–6.2)	Q5 (≥6.3)	
		(*n* = 1,586)	(*n* = 1,484)	(*n* =1,554)	(*n* = 1,596)	(*n* = 1,576)	
Female (*n* = 7,796)	Model 1^a^	Reference	1.08 (0.91, 1.27)	0.89 (0.75, 1.05)	0.79 (0.66, 0.93)	0.77 (0.64, 0.91)	0.0002
	Model 2^b^	Reference	1.13 (0.95, 1.34)	0.92 (0.78, 1.10)	0.82 (0.68, 0.97)	0.81 (0.68, 0.97)	0.0003
	Model 3^c^	Reference	1.14 (0.94, 1.37)	0.90 (0.74, 1.09)	0.81 (0.67, 0.98)	0.83 (0.67, 1.02)	0.0024

a Adjusted for age, race/ethnicity;

b adjusted for age, race/ethnicity, smoking status, drinking status, hypertension status and diabetes status;

c *adjusted for age, race/ethnicity, smoking status, drinking status, hypertension status, diabetes status, creatinine, STC, glucose, BMI, HDL-C, STG, serum calcium, magnesium intake, zinc intake, fiber intake*.

Compared to Q1, adjusted ORs in Q2–Q5 of the total vitamin D intake were 0.83 (95% CI, 0.69–0.98), 0.69 (95% CI, 0.58–0.83), 0.66 (95% CI, 0.55–0.79), and 0.59 (95% CI, 0.48–0.71), respectively, with *p* for trend of < 0.0001 in males. In females, compared to Q1, the adjusted ORs of HU were 0.80 (95% CI, 0.65–0.98) for Q5 (those consuming 19.6 mcg or greater), and the *p* for trend was 0.0076. More detailed information is presented in Table 5.

**Table 5:** Adjusted odds ratios of hyperuricemia among participants associated with total vitamin D intake.

		**Total vitamin D intake (mcg/day)**					***p* for trend**
		Q1 (<1.3)	Q2 (1.3–3.4)	Q3 (3.5–6.8)	Q4 (6.9–14.6)	Q5 (≥14.7)	
		(*n* = 1,515)	(*n* = 1,617)	(*n* = 1,606)	(*n* =1,598)	(*n* = 1,591)	
Male (*n* = 7,927)	Model 1^a^	Reference	0.86 (0.73, 1.01)	0.75 (0.64, 0.89)	0.71 (0.60, 0.84)	0.62 (0.52, 0.73)	<0.0001
	Model 2^b^	Reference	0.85 (0.72, 1.00)	0.72 (0.61, 0.85)	0.69 (0.59, 0.82)	0.59 (0.49, 0.70)	<0.0001
	Model 3^c^	Reference	0.83 (0.69, 0.98)	0.69 (0.58, 0.83)	0.66 (0.55, 0.79)	0.59 (0.48, 0.71)	<0.0001
		Q1 (<1.2)	Q2 (1.2–3.2)	Q3 (3.3–7.2)	Q4 (7.3–19.5)	Q5 (≥19.6)	
		(*n* = 1,487)	(*n* = 1,579)	(*n* =1,609)	(*n* =1,557)	(*n* = 1,564)	
Female (*n* = 7,796)	Model 1^a^	Reference	0.98 (0.82, 1.16)	0.78 (0.65, 0.93)	0.74 (0.62, 0.89)	0.71 (0.60, 0.85)	<0.0001
	Model 2^b^	Reference	1.01 (0.85, 1.21)	0.80 (0.67, 0.96)	0.77 (0.64, 0.92)	0.77 (0.64, 0.92)	0.0001
	Model 3^c^	Reference	1.01 (0.84, 1.22)	0.82 (0.68, 1.33)	0.83 (0.68, 1.02)	0.80 (0.65, 0.98)	0.0076

a Adjusted for age, race/ethnicity;

b adjusted for age, race/ethnicity, smoking status, drinking status, hypertension status and diabetes status;

c *adjusted for age, race/ethnicity, smoking status, drinking status, hypertension status, diabetes status, creatinine, STC, glucose, BMI, HDL-C, STG, serum calcium, magnesium intake, zinc intake, fiber intake*.

In males, compared with the supplemental vitamin D non-user, the adjusted odds ratios (ORs) were 0.77 (95% CI, 0.65–0.92) among those consuming <15 mcg supplemental vitamin D daily, and the *p* for trend was 0.0268. Nevertheless, there was no significant relationship between supplemental vitamin D intake and HU in females, after adjusting for all confounding factors, as is shown in Table 6. All model fitness was assessed by a likelihood ratio test (*p* < 0.0001).

**Table 6:** Adjusted odds ratios of hyperuricemia among participants associated with supplemental vitamin D intake.

		**Supplemental vitamin D intake (mcg/day)**				***p* for trend**
		Non-user	<15	15–100	≥100	
		(*n* = 6109)	(*n* = 1046)	(*n* = 723)	(*n* = 49)	
Male (*n* = 7,927)	Model 1^a^	Reference	0.76 (0.64, 0.90)	0.87 (0.72, 1.06)	0.61 (0.28, 1.30)	0.0068
	Model 2^b^	Reference	0.73 (0.62, 0.87)	0.83 (0.69, 1.01)	0.58 (0.27, 1.25)	0.0013
	Model 3^c^	Reference	0.77 (0.65, 0.92)	0.89 (0.73, 1.09)	0.65 (0.30, 1.43)	0.0268
		Non-user	<15	15–100	≥100	
		(*n* = 5,341)	(*n* = 1,004)	(*n* = 1,350)	(*n* = 101)	
Female (*n* = 7,796)	Model 1^a^	Reference	0.83 (0.70, 0.98)	0.82 (0.70, 0.95)	0.68 (0.42, 1.10)	0.0100
	Model 2^b^	Reference	0.84 (0.71, 1.00)	0.85 (0.73, 0.99)	0.71 (0.43, 1.16)	0.0089
	Model 3^c^	Reference	0.95 (0.79, 1.14)	0.89 (0.75, 1.05)	0.68 (0.41, 1.16)	0.0804

a Adjusted for age, race/ethnicity;

b adjusted for age, race/ethnicity, smoking status, drinking status, hypertension status and diabetes status;

c *adjusted for age, race/ethnicity, smoking status, drinking status, hypertension status, diabetes status, creatinine, STC, glucose, BMI, HDL-C, STG, serum calcium, magnesium intake, zinc intake, fiber intake*.

A sensitivity analysis was undertaken using the second 24-h dietary recall data from 2007 to 2014. Thirteen thousand nine hundred seventy eight adults (6,890 male and 7,088 female) were included in sensitivity analysis. We used the mean of the nutrient intake from the two dietary recalls and adjusted for the same covariates of the primary analyses. By and large, the relationships between the intake of dietary vitamin D, supplemental vitamin D, and total vitamin D with HU risk were not altered.

## Discussion

In this large population-based study, a significant negative association between serum 25(OH) D (Q1 vs. Q2, Q4), dietary vitamin D intake (Q1 vs. Q3–Q5), supplemental vitamin D intake (non-user vs. <15 mcg/day), and total vitamin D intake (Q1 vs. Q2–Q5) with the risk of HU was found in men. We observed inverse associations between serum 25(OH) D (Q1 vs. Q3), dietary vitamin D intake (Q1 vs. Q4), and total vitamin D intake (Q1 vs. Q5) with HU in women among US adults.

To the best of our knowledge, this is the first study revealing the association of serum 25(OH) D, dietary vitamin D intake, supplemental vitamin D intake, and total vitamin D intake with HU in both male and female of US adults, and the largest population-based study using a nationally representative sample. Some studies have reported that serum 25(OH) D was associated with the metabolic syndrome (14, 15), and vitamin D insufficiency has been found in chronic kidney diseases (12, 27); therefore, we adjusted for metabolic risk factors such as STG, STC, HDL-C, glucose, BMI, hypertension status, and diabetes status and adjusted for creatinine considering renal dysfunction. The previous study has examined that magnesium intake significantly interacted with vitamin D status (28); we adjusted for magnesium intake and also adjusted for the intakes of zinc and fiber for other covariates including age, race/ethnicity, smoking status, drinking status, and serum calcium in multivariate logistic regression models. Coinciding with our results, a research among postmenopausal Chinese Han women found a significant association between the vitamin D insufficiency and elevated uric acid (29). Another similar study among elderly Egyptians found that the low level of vitamin D was significantly associated with high uric acid level (30). However, a study in France which enrolled 192 women ≥65 years revealed that the proportion of women with elevated serum uric acid level was significantly greater in those who received both calcium and vitamin D compared with those who received placebo (31). Major reasons for the inconsistent results can be explained due to the difference in age of the research participants and different countries. Our study also did not observe associations of the supplemental vitamin D intake with HU risk in females.

The Food and Nutrition Board (FNB) committee noted that serum levels of 50 nmol/L or more are sufficient for most people and serum concentrations >125 nmol/L can be associated with adverse effects. In our study, the adjusted OR was 0.78 among those serum 25(OH) D levels of 41.8–55.4 nmol/L, compared with respondents' serum levels <41.8 nmol/L for males, and OR was 0.80 among those serum 25(OH) D levels of 55.4–69.3 nmol/L, compared with respondents' serum levels <39.8 nmol/L for females. The recommended dietary allowance (RDA) for vitamin D was 15 mcg/day for US adults aged 19–70 years and 20 mcg/day for aged 70 years and above. In our results, the OR was 0.80 among those consuming more than 19.6 mcg vitamin D daily, compared with respondents consuming <1.2 mcg vitamin D daily for females. Our results suggested that adequate vitamin D may have a potential function for preventing or decreasing the risk of HU. The underlying mechanism for the association between vitamin D status and the risk of HU was not completely explained, and several hypotheses have been proposed. First, previous studies showed that serum 25-hydroxy vitamin D level insufficiency can activate parathyroid to induce the release of parathyroid hormone. Meanwhile, HU and gout have become more frequent in patients with hyperparathyroidism, and parathyroidectomy can reduce serum uric acid levels in these cases. Increased parathyroid hormone levels are thought to reduce uric acid excretion in the kidney. Furthermore, several studies showed a significant association between parathyroid hormone and serum uric acid levels. Previous clinical trials of 1,637 postmenopausal women found that parathyroid hormone increased the incidence of hyperuricemia in a dose-response fashion (18, 19), and serum uric acid levels decrease after cessation of treatment (19). A nationally representative population study among 8,316 participants, from the US and male and female, indicated that serum uric acid levels and the frequency of hyperuricemia increased with increasing parathyroid hormone levels (20). Therefore, serum 25-hydroxy vitamin D is likely inversely associated with elevated serum uric acid levels. Further studies are required to investigate the biological mechanism between vitamin D status and HU.

In addition, a systematic review found that moderate to high doses of vitamin D supplementation may reduce cardiovascular diseases (32), and it was reported that higher vitamin D intake is associated with lower cardiovascular disease risk in US males (10). Meanwhile, numerous studies have demonstrated that elevated serum uric acid level was a risk factor for cardiovascular diseases and independently associated with cardiovascular mortality (33–37). A large prospective long study of 83,683 Austrian males found that serum uric acid was independently related to mortality from cardiovascular disease, suggesting the clinical importance of monitoring and intervention based on serum uric acid, which was easily and routinely measured (37). Our results show significantly higher levels of STC, STG, and glucose and a lower level of HDL in patients with HU than those participants without HU. Previous studies show that serum uric acid levels were positively associated with triglycerides and systolic blood pressure and negatively associated with HDL cholesterol (38). Thus, it is necessary to clarify the effect of vitamin D supplementation on HU and consequently the effect of serum uric acid, lowering treatment on the prevention of cardiovascular diseases.

The main strengths of our study are as follows. Firstly, this is the first study that directly investigated the relationship between serum 25(OH) D intake, dietary vitamin D intake, supplemental vitamin D intake, total vitamin D intake, and the risk of HU in both male and female, based on a large (15,723 subjects) and nationally representative sample among US adults. Secondly, we adjusted for a considerable number of potential confounding variables. Thirdly, the use of trained staff to evaluate the main information of the research object and conduct interviews in accordance with a standardized program has improved the accuracy and effectiveness of the data. Nevertheless, our study has several limitations. Firstly, our study was a cross-sectional design study, which limited the definition of the causal correlational relationship between vitamin D status and HU; further prospective longitudinal studies would be important to support these conclusions. Secondly, although we adjusted several main covariates in the analysis, the associations reported may partially be due to the potential confusion by other unobserved variables and residual confounding. Thirdly, the data on sun exposure were not available. However, we used a direct measure of serum 25(OH) D, which reflected cumulative sun exposure and dietary vitamin D intake, and we also used the data of supplemental vitamin D intake. Finally, further future prospective studies and clinical trials are needed to investigate the underlying mechanisms of those associations.

## Conclusions

Our findings indicated that the serum 25(OH) D, dietary vitamin D intake, supplemental vitamin D intake, and total vitamin D intake were inversely related to risk of HU in men. We observed a lower risk of HU with higher serum 25(OH) D, dietary vitamin D intake, and total vitamin D intake and no association between supplemental vitamin D intake and the risk of HU in women among US adults, independent of some major confounding factors.

## Data Availability Statement

The raw data supporting the conclusions of this article will be made available by the authors, without undue reservation.

## Author Contributions

Y-YZ, H-BQ, and J-WT designed the study and wrote the manuscript. Y-YZ analyzed and interpreted the data. All authors read and approved the final manuscript.

## Conflict of Interest

The authors declare that the research was conducted in the absence of any commercial or financial relationships that could be construed as a potential conflict of interest.
